# Hypothesis‐Generating Single‐Cell Analysis of the Endometrial Cancer Immune Microenvironment With Complementary Multiomics Evidence

**DOI:** 10.1155/ijog/5125648

**Published:** 2026-10-18

**Authors:** Xiaolan Ouyang, Xiaoli Guo, Xiaoxi Yang, Juan Wen

**Affiliations:** ^1^ Chengdu Women′s and Children′s Central Hospital School of Medicine University of Electronic Science and Technology of China Chengdu China uestc.edu.cn

**Keywords:** anti–PD-1 response, endometrial cancer, immune microenvironment, microbiome, qRT-PCR, single-cell RNA sequencing, TCGA-UCEC

## Abstract

**Background:**

Endometrial cancer (EC) has a heterogeneous immune microenvironment, and response‐associated biology remains incompletely characterized. We used the retained EC single‐cell dataset to describe immune‐cell states and complemented it with TCGA‐UCEC, independent gut microbiome cohorts, and RL95‐2 proof‐of‐concept experiments. All responder versus nonresponder scRNA‐seq comparisons are descriptive and hypothesis generating because only one sample was available per clinical response group.

**Methods:**

Single‐cell RNA sequencing (scRNA‐seq) data from EC patients undergoing anti–PD‐1 immunotherapy (GEO: GSE251923; one responder, GSM7990051, and one nonresponder, GSM7990052; 17,546 cells retained after quality control) were analyzed using Scanpy, Leiden clustering, and over‐representation analysis with GO biological process, KEGG, and MSigDB Hallmark gene sets using Enrichr with Benjamini–Hochberg correction. Bulk RNA‐seq from TCGA‐UCEC (554 tumor and 35 normal samples) was used for complementary transcriptomic analysis. Gut microbiome 16S rRNA datasets (SRA: PRJNA897842 and PRJNA833670) were analyzed as independent cohorts, and RL95‐2 qRT‐PCR experiments were interpreted as proof‐of‐concept.

**Results:**

Ten major cell populations were identified in the EC immune microenvironment. In this single responder/nonresponder sample pair, the nonresponder sample showed patterns consistent with higher M2‐associated macrophage marker expression, increased T‐cell checkpoint/exhaustion marker expression, and lower cytotoxic activity scores. These observations are descriptive and cannot establish patient‐level significance. TCGA‐UCEC analyses and the independent microbiome and RL95‐2 experiments provide complementary, hypothesis‐generating evidence; no unverified classifier‐performance estimate is reported.

**Conclusions:**

This study provides a hypothesis‐generating description of immune patterns in one EC responder and one nonresponder sample, complemented by TCGA‐UCEC, independent microbiome datasets, and RL95‐2 proof‐of‐concept experiments. The findings nominate candidate immune, hormonal, and microbiome‐associated pathways for further investigation but do not establish patient‐level response differences, causality, or clinically validated biomarkers.

## 1. Introduction

Endometrial cancer (EC) is a common gynecologic malignancy with substantial molecular and clinical heterogeneity. The tumor immune microenvironment influences disease progression and may shape response to immunotherapy, yet the cellular programs that distinguish treatment‐response states remain incompletely defined.

The endometrial immune microenvironment is a specialized tissue niche that undergoes extensive remodeling across the menstrual cycle and in disease [1]. Uterine natural killer (uNK) cells, macrophages, T‐cell subsets, and stromal cells coordinate tissue remodeling, inflammatory control, and tolerance at the maternal‐fetal interface [2, 3]. These same lineages and their signaling programs can be altered in malignant endometrial tissue, motivating single‐cell characterization of EC immune states.

Endometrial macrophages exhibit functional plasticity between proinflammatory and anti‐inflammatory states [4]. T‐cell subsets, including CD4+ helper, CD8+ cytotoxic, and regulatory T cells, shape local immune tolerance and antitumor activity [5]. Dendritic cells bridge innate and adaptive immunity and can influence T‐cell priming and regulatory programs [6]. Defining the balance among these populations is important for interpreting response‐associated EC biology.

Single‐cell RNA sequencing (scRNA‐seq) enables high‐resolution characterization of complex tissue microenvironments [7]. Studies of human endometrium and decidua have revealed cellular diversity and dynamic transcriptional states at the maternal‐fetal interface [8]. Applying the same resolution to EC can identify candidate immune‐cell programs and pathways for subsequent validation.

LIF (leukemia inhibitory factor) and its receptor LIFR constitute a cytokine axis involved in stromal and immune regulation in the endometrium [9]. LIF signaling through LIFR/gp130‐JAK1‐STAT3 supports stromal differentiation and immune‐cell function. Its relevance to EC response biology remains a question for future directly replicated studies.

An emerging body of evidence indicates that the gut microbiome can modulate systemic inflammatory tone and immune homeostasis. Dysbiosis, altered short‐chain fatty‐acid production, and microbial products such as lipopolysaccharide may influence innate immune signaling [10, 11]. In the present study, microbiome findings are analyzed as an independent exploratory evidence stream and are not treated as within‐patient mechanistic evidence for the EC single‐cell observations.

In this study, we performed a multiomics, hypothesis‐generating analysis centered on the EC immune microenvironment, integrating scRNA‐seq from two EC samples receiving anti–PD‐1 immunotherapy (GSE251923; one responder and one nonresponder; 17,546 cells), bulk TCGA‐UCEC transcriptomics, and independent gut microbiome datasets (PRJNA897842 and PRJNA833670). Because the microbiome and scRNA‐seq cohorts are nonoverlapping, microbiome findings are interpreted independently and not as within‐patient mechanistic evidence. RL95‐2 qRT‐PCR experiments provide a separate proof‐of‐concept assessment of stimulus‐responsive gene expression.

## 2. Methods

### 2.1. Data Source and Study Population

Single‐cell RNA‐seq data were obtained from the Gene Expression Omnibus (GEO) under accession GSE251923 and comprised two EC samples from patients receiving anti–PD‐1 immunotherapy: GSM7990051 (responder) and GSM7990052 (nonresponder). Thus, there was one biological sample per response group. Bulk RNA‐seq data for uterine corpus endometrial carcinoma (TCGA‐UCEC) were retrieved from the NCI Genomic Data Commons, including 554 tumor and 35 normal adjacent‐tissue samples. Gut microbiome sequencing data were drawn from the independent SRA cohorts described below. All datasets analyzed are publicly available and deidentified.

### 2.2. Single‐Cell RNA‐Seq Data Processing

Single‐cell RNA‐seq data were processed using Scanpy (v1.9+). Quality control filters were applied to retain cells with at least 200 detected genes and mitochondrial gene percentage below 20%. After QC, a total of 17,546 cells were retained for downstream analysis. Data were normalized to library size with a scaling factor of 10,000 followed by log1p transformation. Highly variable genes (Top 2000) were selected using the seurat_v3 flavor. Principal component analysis (PCA) was performed on the highly variable genes, and the Top 30 principal components were used for UMAP visualization and Leiden clustering (resolution optimized based on silhouette score). Cell type annotation was performed using a differential expression (DE)‐based approach: for each Leiden cluster, the Top 15 differentially expressed genes were identified via rank_genes_groups (Wilcoxon test) and matched against a curated marker gene library covering 10 distinct cell types sourced from PanglaoDB, CellMarker 2.0, and published literature. Cluster‐level identity was assigned based on the highest marker overlap count (minimum overlap threshold of 1). Clusters with no marker match were assigned temporary names based on their top differentially expressed genes and subsequently reannotated after marker library expansion. The final annotation resolved the following cell populations: B cells, Cl0_MDK + FBLN1, Cl1_WFDC2 + SLPI, endothelial cells, epithelial cells, fibroblasts, macrophages, mast cells, plasma cells, T cells. Responder‐versus‐nonresponder comparisons were treated as descriptive because only one biological sample was available per group.

### 2.3. DE and Pathway Enrichment

At the single‐cell level, exploratory DE was performed per cell type using the Wilcoxon rank‐sum test implemented in Scanpy rank_genes_groups between the responder and nonresponder cell sets, using |log2 fold change| > 0.25 and Benjamini–Hochberg‐adjusted *p* < 0.05 as gene‐prioritization thresholds. Because cells from the same donor are not independent biological replicates and only one donor was available per group, these cell‐level *p* values are anticonservative for patient‐level inference; the resulting genes are therefore interpreted as exploratory candidates rather than confirmatory condition‐associated DEGs [12, 13]. A donor‐level pseudobulk comparison could not be performed because biological replication was unavailable. For TCGA‐UCEC, tumor (*n* = 554) and normal (*n* = 35) samples were compared with multiple‐testing correction as described above. Pathway enrichment used gseapy Enrichr as an over‐representation analysis (ORA) against GO biological process, KEGG, and MSigDB Hallmark gene sets; the submitted gene universe consisted of genes retained in the relevant analysis, and pathway *p* values were corrected using the Benjamini–Hochberg procedure, with adjusted *p* < 0.05 used for prioritization.

### 2.4. Immune Microenvironment Characterization

The immune microenvironment was characterized using signature‐based scoring. Immune checkpoint molecule expression (including PDCD1, CTLA4, LAG3, TIGIT, HAVCR2) was quantified using normalized expression values. Cytotoxic activity was assessed via the geometric mean expression of GZMA, GZMB, PRF1, and IFNG. Macrophage polarization status was evaluated using M1 (TNF, IL1B, CXCL10, NOS2) and M2 (CD163, MRC1, MSR1, ARG1) signature scores. Immune infiltration was estimated using signature‐based scoring across cell types, and the ESTIMATE‐derived immune and stromal scores were computed for the TCGA‐UCEC cohort.

### 2.5. Survival and Clinical Correlation

Overall survival analysis was described using the Kaplan–Meier method, with patient stratification based on median expression of signature scores or selected gene markers and log‐rank testing between strata.

### 2.6. Quantitative RT‐PCR Proof‐of‐Concept Analysis

Gene expression experiments were performed in RL95‐2 (ATCC HTB‐109), a human endometrial carcinoma cell line, cultured in DMEM/F12 supplemented with 10% FBS at 37°C with 5% CO_2_. Three separate stimulation conditions were used: estrogen deprivation with charcoal‐stripped serum (48 h) to assess ESR1; TGF‐*β*1 (10 ng/mL, 48 h) to examine stimulus‐responsive expression of KLRB1, CD56, EOMES, and FOXP3; and LPS (100 ng/mL, 24 h) to examine CD68. Vehicle‐treated cells served as controls. RNA extraction, cDNA synthesis, and qRT‐PCR were performed as described, with GAPDH used for normalization and three independent experiments analyzed by two‐tailed Student′s *t*‐test. Because RL95‐2 is an epithelial carcinoma cell line and lacks uNK cells, macrophages, and T cells, these experiments are interpreted as proof‐of‐concept gene‐regulatory responses and not as validation of lineage‐specific immune‐cell biology. The stimulation conditions were applied separately and do not reproduce an integrated multicellular microenvironment.

## 3. Results

### 3.1. Single‐Cell Transcriptomic Landscape of the EC Immune Microenvironment

After QC filtering, 17,546 cells were retained and embedded in UMAP space, identifying 10 distinct cell populations: B cells (5.1%), fibroblasts (16.7%), epithelial cells (14.7%), T cells (10.7%), macrophages (3.9%), and other lineages (Figures 1A–C). Cell type composition analysis showed descriptive differences in immune cell proportions between responder and nonresponder groups, with lineage‐defining markers confirming all cluster assignments (Figures 1D–F). Because *n* = 1 biological sample was available per condition, these composition differences are not interpreted as patient‐level statistically significant effects.

**Figure 1:**
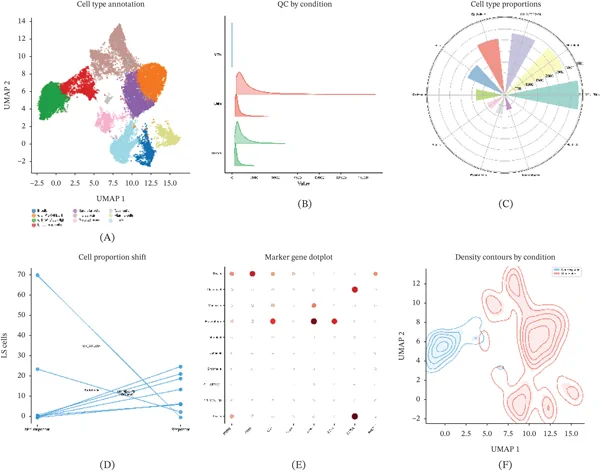
Single‐cell transcriptomic landscape and cellular composition of the endometrial cancer microenvironment. (A) UMAP visualization of single cells, colored by annotated cell population. (B) Distributions of quality‐control metrics by condition. (C) Polar plot summarizing the distribution of cells across annotated cell populations. (D) Comparison of cell type proportions between the nonresponder and responder samples. Each line connects the proportions of the same cell population across the two conditions; the vertical axis indicates the percentage of cells. (E) Dot plot of marker gene expression across annotated cell populations. Dot size represents the percentage of cells expressing each gene, and color intensity indicates the mean expression level. (F) UMAP density contours showing the distributions of cells from the responder and nonresponder samples. The scRNA‐seq dataset comprises one biological sample per condition; therefore, between‐condition comparisons are descriptive and exploratory, and no patient‐level statistical inference is made.

### 3.2. Immune Cell Subtype Characterization and Transcriptional Heterogeneity

Subclustering resolved transcriptional heterogeneity within immune lineages and suggested differences in activation, exhaustion, and effector programs (Figures 2A,B). In the single nonresponder sample, checkpoint/exhaustion markers (PDCD1, CTLA4, LAG3, TIGIT, HAVCR2) appeared higher in selected T‐cell subsets; cytotoxic activity scores appeared lower; and macrophage profiles showed greater expression of M2‐associated markers (CD163, MRC1, MSR1) relative to the single responder sample (Figures 2C–F). These patterns are descriptive and hypothesis generating; cell‐level tests do not provide patient‐level inference in the absence of biological replication.

**Figure 2:**
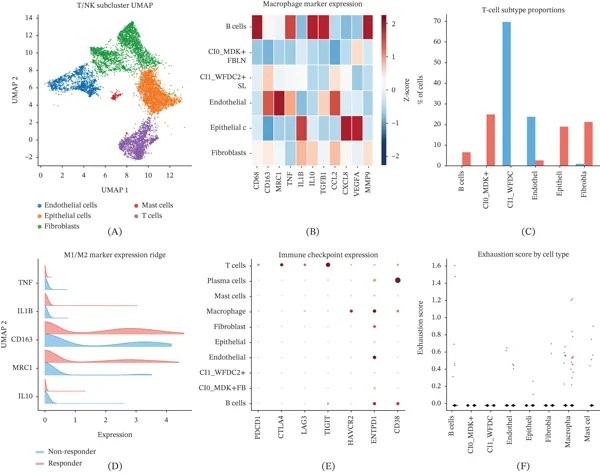
Immune cell subtype characterization and transcriptional heterogeneity. (A) Subclustering UMAP of major immune lineages (T cells, NK cells, myeloid cells) revealing transcriptional heterogeneity and distinct functional states within each lineage. (B) Heatmap of functional gene signatures across immune subtypes. Hierarchical clustering groups subtypes by shared functional programs including activation, exhaustion, and effector modules. (C) Expression analysis of immune checkpoint and exhaustion markers (PDCD1, CTLA4, LAG3, TIGIT, HAVCR2) across T cell subsets, comparing responder and nonresponder conditions. (D) Cytotoxic activity signature scoring across immune subtypes. GZMA, GZMB, PRF1, and IFNG expression levels were summarized as geometric mean per cell. (E) Macrophage M1/M2 polarization analysis. M1 markers (TNF, IL1B, CXCL10) and M2 markers (CD163, MRC1, MSR1) were compared across conditions. (F) Density distribution (ridge or raincloud plot) of signature scores stratified by condition, illustrating the heterogeneity of immune functional states within each cell compartment. For scRNA‐seq panels comparing responder and nonresponder, *n* = 1 biological sample per condition; comparisons are descriptive and no patient‐level inferential significance is claimed. Cell‐level statistics, where displayed, are interpreted as exploratory.

### 3.3. DE and Pathway Enrichment in EC Immune Cells

Exploratory cell‐level analysis prioritized 6975 genes meeting the prespecified Wilcoxon/adjusted‐*p* thresholds (6433 higher and 542 lower in the nonresponder cell sets), with macrophages contributing the largest number of prioritized genes (Figure 3B). Because the analysis treats cells from one donor per condition as observations, these counts likely overstate inferential precision and should not be interpreted as 6975 independently validated condition‐associated DEGs [12, 13]. The ranked genes and enrichment results are used for hypothesis generation only.

**Figure 3:**
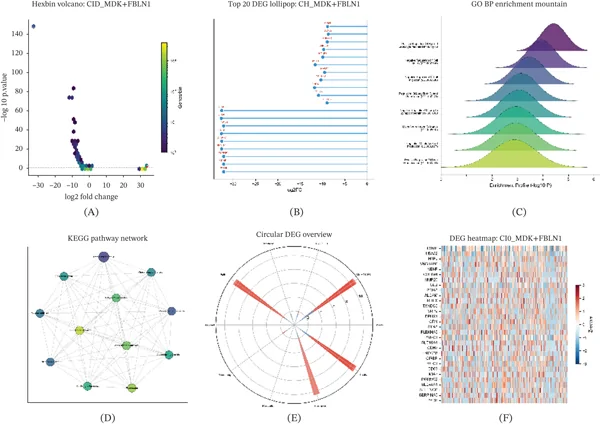
Differential expression and pathway enrichment in EC immune cells. (A) Volcano plot of differentially expressed genes (responder vs. nonresponder). Log2 fold change plotted against −log10 adjusted *p* value. Genes meeting the exploratory thresholds (|log2*F* *C*| > 0.25, adj.*p* < 0.05) are highlighted. (B) Per‐cell‐type differential expression summary bar chart or lollipop plot. The number of upregulated and downregulated DEGs is shown for each identified cell type. (C) Pathway enrichment dotplot or bubble chart of GO Biological Process and KEGG pathways. Dot size represents gene count and color intensity indicates enrichment significance. (D) Hexbin volcano or circular DE plot providing enhanced visualization of differential expression results with density information. (E) Diverging bar or ranked lollipop plot of the top DEGs sorted by absolute fold change. Upregulated genes are displayed in one direction and downregulated genes in the opposite. (F) Signature score comparison between conditions across immune cell types. Composite scores for curated gene signatures (immune, oncogenic, metabolic) are shown. For scRNA‐seq panels comparing responder and nonresponder, *n* = 1 biological sample per condition; comparisons are descriptive and no patient‐level inferential significance is claimed. Cell‐level statistics, where displayed, are interpreted as exploratory.

### 3.4. Bulk Transcriptomic Analysis and Clinical Implications in TCGA‐UCEC

TCGA‐UCEC bulk RNA‐seq analysis (554 tumor vs. 35 normal) identified 6511 upregulated and 6502 downregulated DEGs, with PRAME (log2FC = 4.9, *p* = 2.1 × 10 − 15) and DES (log2FC = −6.9, *p* = 1.1 × 10 − 22) as the top differentially expressed genes. PCA, DE, immune composition, immune checkpoint, survival, clinical factor, cross‐platform fold change, pathway activity, and immune signature analyses are summarized in Figures 4 and 5.

**Figure 4:**
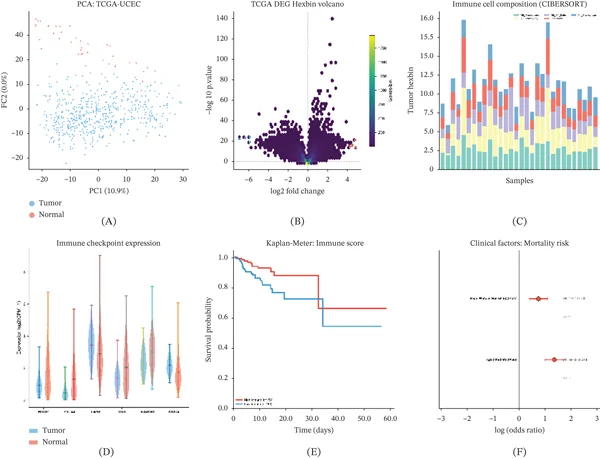
TCGA‐UCEC bulk transcriptomic and clinical analyses. (A) PCA visualization of TCGA‐UCEC tumor and normal samples. (B) Hexbin volcano plot of TCGA‐UCEC differential expression. (C) Estimated immune‐cell composition across TCGA‐UCEC samples. (D) Distribution of selected immune‐checkpoint expression measures. (E) Kaplan–Meier survival curves stratified by immune score. (F) Forest‐plot summary of clinical factors associated with mortality risk.

**Figure 5:**
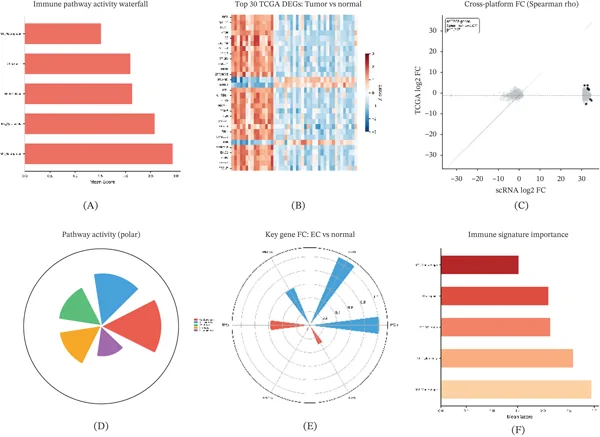
Additional TCGA‐UCEC and cross‐platform transcriptomic analyses. (A) Ranked immune‐pathway activity scores. (B) Heatmap of the top TCGA‐UCEC tumor‐versus‐normal DEGs. (C) Cross‐platform fold‐change comparison between scRNA‐seq and TCGA‐UCEC results, with correlation summarized in the panel. (D) Polar visualization of pathway‐activity scores. (E) Fold‐change summary for selected key genes in EC versus normal tissue. (F) Relative importance of selected immune signatures. These panels present exploratory EC/TCGA analyses only.

### 3.5. Gut Microbiota Alterations and Diagnostic Implications in EC

Alpha diversity appeared reduced in EC patients, with Shannon and Chao1 differences shown in Figure 6A (*p* = 0.004 and *p* = 0.008, respectively). PCoA visualized sample‐level compositional patterns, whereas the retained PERMANOVA was not significant (*p* = 0.593; Figure 6B). At the phylum level, Bacillota and Bacteroidota were dominant components of both groups (Figure 6C). Genus‐level abundance patterns, differential‐taxon analysis, and a microbial correlation network are shown in Figure 6D–E; these analyses suggested differences involving genera including Bilophila, Roseburia, and Faecalicatena. The microbiome samples were obtained from cohorts independent of GSE251923; therefore, these findings cannot be linked to the scRNA‐seq immune phenotypes at the individual‐patient level.

**Figure 6:**
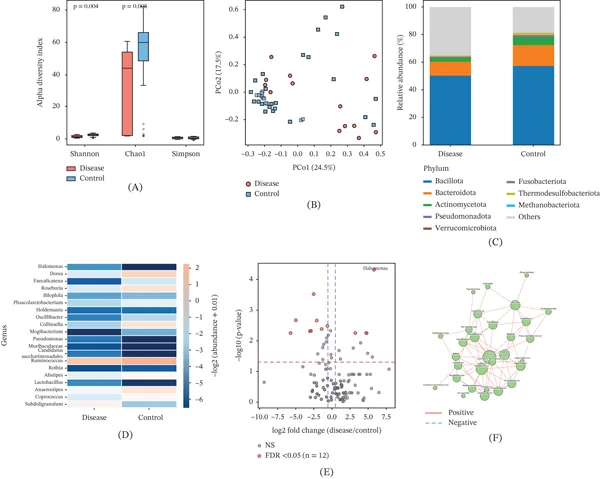
Gut microbiota alterations in endometrial cancer. (A) Alpha‐diversity boxplots (Shannon, Chao1, and Simpson indices) comparing disease and control groups; *p* values displayed in the panel are based on between‐group testing. (B) Bray–Curtis PCoA ordination showing group‐level compositional patterns. (C) Phylum‐level relative abundance, including Bacillota and Bacteroidota. (D) Genus‐level abundance heatmap comparing disease and control groups. (E) Differential‐taxon volcano plot with FDR‐based significance annotation. (F) Microbial correlation network showing positive and negative associations among genera.

### 3.6. Exploratory Microbiome‐Associated Diagnostic Modeling

Figure 7 summarizes exploratory associations between microbial alpha diversity and available clinical characteristics, together with a microbiome‐based random forest classifier and feature‐importance analysis. The random forest model yielded an AUC of 0.865 (Figure 7D). Because these microbiome data were obtained from cohorts independent of GSE251923, this analysis does not represent a within‐patient host‐microbiome model and should be interpreted as exploratory.

**Figure 7:**
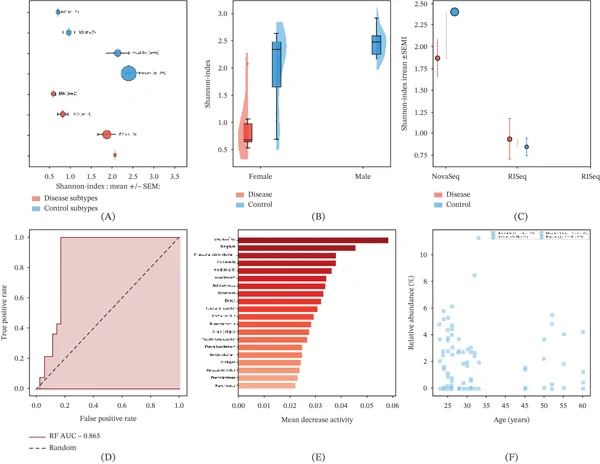
Microbiome‐associated clinical features and exploratory diagnostic modeling in EC. (A) Stratified Shannon‐diversity summaries across disease and control subgroups. (B) Shannon‐diversity distribution stratified by sex. (C) Group‐level Shannon‐index summaries across available clinical strata. (D) Receiver operating characteristic curve for the exploratory microbiome‐based random forest classifier (AUC = 0.865). (E) Random forest feature‐importance ranking of microbial taxa. (F) Scatter plot illustrating the relationship between age and relative abundance for selected microbial features. These analyses are based on the microbiome cohort and do not represent within‐patient integration with GSE251923 scRNA‐seq data

### 3.7. Quantitative RT‐PCR Proof‐of‐Concept Analysis in RL95‐2 Cells

To examine proof‐of‐concept stimulus‐responsive gene expression in vitro, qRT‐PCR was performed on RL95‐2 (ATCC HTB‐109) cells under three stimulation conditions (Figure 8). Under TGF‐*β*1 stimulation, KLRB1 (7.0 ± 0.74‐fold reduction, *p* < 0.001), CD56 (7.75 ± 0.71‐fold reduction, *p* < 0.001), and EOMES (8.67 ± 0.85‐fold reduction, *p* < 0.001) were suppressed, whereas FOXP3 was 2.73 ± 0.42‐fold elevated (*p* < 0.001). Under LPS stimulation, CD68 was 2.46 ± 0.38‐fold upregulated (*p* < 0.001). Under estrogen deprivation (charcoal‐stripped serum), ESR1 was 11.3 ± 1.38‐fold downregulated (*p* < 0.001), consistent with an estrogen‐deprived epithelial context. All findings are expressed as fold change versus vehicle control (mean ± SEM, *n* = 3 independent experiments) and demonstrate stimulus‐responsive expression in RL95‐2 cells but do not validate immune‐cell‐specific responses observed by scRNA‐seq.

**Figure 8:**
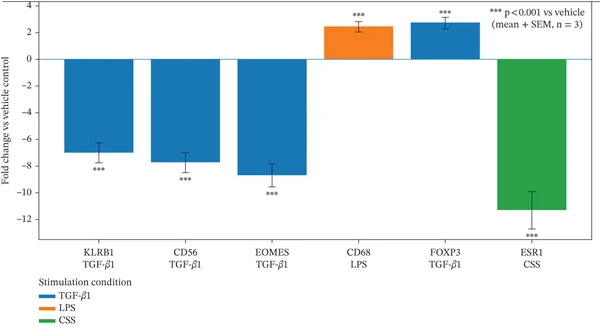
Quantitative RT‐PCR proof‐of‐concept analysis in RL95‐2 (ATCC HTB‐109) cells. Relative mRNA expression of KLRB1, CD56, EOMES, CD68, FOXP3, and ESR1 was normalized to GAPDH. TGF‐*β*1 (10 ng/mL, 48 h) was used for KLRB1, CD56, EOMES, and FOXP3; LPS (100 ng/mL, 24 h) was used for CD68; and charcoal‐stripped serum (CSS, 48 h) was used for ESR1. Vehicle‐treated cells served as controls. Error bars represent SEM from three independent experiments. Significance:  ^∗∗∗^ *p* < 0.001 by two‐tailed Student′s *t*‐test. KLRB1, CD56, and EOMES were suppressed under TGF‐*β*1, FOXP3 was elevated under TGF‐*β*1, CD68 was elevated under LPS, and ESR1 was downregulated under estrogen deprivation. These results demonstrate stimulus‐responsive gene expression in RL95‐2 cells and do not constitute lineage‐specific immune‐cell validation.

## 4. Discussion

This study provides a hypothesis‐generating multiomics analysis centered on the EC immune microenvironment. The scRNA‐seq component profiles 17,546 cells from only two patients (one responder and one nonresponder), whereas TCGA‐UCEC and the microbiome datasets provide complementary information from separate cohorts. The apparent M2‐associated macrophage pattern and T‐cell exhaustion pattern are descriptive observations in this sample pair rather than patient‐level inferential findings. The microbiome and RL95‐2 results are interpreted as independent, complementary evidence streams.

In the single nonresponder sample, macrophages showed greater expression of M2‐associated markers such as CD163, MRC1, and MSR1, whereas selected T‐cell subsets showed higher checkpoint‐marker expression and lower cytotoxic activity scores than the single responder sample. These observations generate a biologically plausible hypothesis of coordinated myeloid and T‐cell immunosuppression, but the absence of biological replication prevents separation of treatment‐response biology from interindividual variability, technical sampling effects, or batch effects. The 6975 genes prioritized by cell‐level Wilcoxon testing should therefore be regarded as exploratory candidates rather than confirmatory DEGs, consistent with evidence that single‐cell DE methods that ignore biological replicates can generate anticonservative results [12, 13].

The RL95‐2 experiments provide proof‐of‐concept evidence that selected transcripts respond to TGF‐*β*1, LPS, or estrogen deprivation in an endometrial carcinoma epithelial context (Figure 8). They do not validate suppression of NK‐lineage programs in uNK cells, macrophage polarization, or Treg biology because RL95‐2 cells are not immune cells. Furthermore, the stimuli were applied separately rather than as a combined microenvironmental model. Primary NK‐cell, monocyte/macrophage, and endometrial tissue experiments will therefore be needed for lineage‐specific mechanistic validation.

The microbiome findings should also be interpreted cautiously. Reduced diversity and shifts involving Bilophila, Roseburia, and Faecalicatena are compatible with hypotheses linking microbial dysbiosis, endotoxin exposure, and inflammatory signaling; however, the microbiome and scRNA‐seq data were obtained from nonoverlapping cohorts. Consequently, the present study cannot demonstrate that gut dysbiosis drives or mechanistically amplifies the immune patterns observed in GSE251923. The proposed microbiome‐TLR4‐NF‐*κ*B‐immune axis is therefore a testable model supported by circumstantial cross‐cohort evidence, requiring validation in paired longitudinal cohorts and experimental intervention studies.

## 5. Limitations

Several limitations merit consideration. First, the scRNA‐seq dataset comprises only two biological samples (one responder and one nonresponder); all between‐condition single‐cell comparisons are therefore descriptive and hypothesis‐generating rather than patient‐level statistical inference. Cell‐level Wilcoxon testing treats cells from the same donor as independent observations and can underestimate biological variance; accordingly, the large number of prioritized genes may partly reflect pseudoreplication, and donor‐level pseudobulk analysis is not possible without biological replication [12, 13]. Second, microbiome and scRNA‐seq measurements were obtained from nonoverlapping cohorts; therefore, direct within‐patient host‐microbiome integration and causal linkage cannot be established. Third, 16S rRNA sequencing has limited taxonomic and functional resolution. Fourth, RL95‐2 is an endometrial carcinoma epithelial cell line and cannot validate uNK‐, macrophage‐, or T‐cell‐specific transcriptional responses; the separate stimulation conditions also do not reproduce the integrated multicellular microenvironment. Finally, the available records do not support reconstruction of additional unreported model parameters or uncertainty estimates, so all retained modeling results are interpreted as exploratory.

## 6. Conclusions

In conclusion, the present study offers a descriptive, hypothesis‐generating view of the EC immune microenvironment based on one responder and one nonresponder scRNA‐seq sample, with complementary TCGA‐UCEC analysis, independent microbiome datasets, and RL95‐2 proof‐of‐concept experiments. The data nominate candidate macrophage, T‐cell, hormonal, and microbiome‐associated pathways for future study but do not establish patient‐level response differences, causal host‐microbiome mechanisms, or clinically validated biomarkers. Larger biologically replicated EC cohorts, paired host‐microbiome datasets, primary immune‐cell experiments, and independently specified model‐validation studies are required to test these hypotheses.

## Author Contributions

All authors contributed to conceptualization, data analysis, manuscript drafting, and critical revision.

## Funding

No funding was received for this manuscript.

## Disclosure

All authors approved the final version.

## Ethics Statement

This study analyzed publicly available, deidentified datasets and commercially available cell lines and did not involve prospective recruitment of human participants or collection of identifiable human specimens. Therefore, additional institutional ethical approval and informed consent were not required.

## Conflicts of Interest

The authors declare no conflicts of interest.

## Data Availability

The data that support the findings of this study are available from the corresponding author upon reasonable request.

## References

[1] Mor G. , Aldo P. , and Alvero A. B. , The Unique Immunological and Microbial Aspects of Pregnancy, Nature Reviews Immunology. (2017) 17, no. 8, 469–482, 10.1038/nri.2017.64, 28627518. 28627518

[2] Moffett A. and Colucci F. , Uterine NK Cells: Active Regulators at the Maternal-Fetal Interface, Journal of Clinical Investigation. (2014) 124, no. 5, 1872–1879, 10.1172/JCI68107, 24789879. PMC4001528 24789879

[3] Vento-Tormo R. , Efremova M. , Botting R. A. , Turco M. Y. , Vento-Tormo M. , Meyer K. B. , Park J. E. , Stephenson E. , Polański K. , Goncalves A. , Gardner L. , Holmqvist S. , Henriksson J. , Zou A. , Sharkey A. M. , Millar B. , Innes B. , Wood L. , Wilbrey-Clark A. , Payne R. P. , Ivarsson M. A. , Lisgo S. , Filby A. , Rowitch D. H. , Bulmer J. N. , Wright G. J. , Stubbington M. J. T. , Haniffa M. , Moffett A. , and Teichmann S. A. , Single-cell Reconstruction of the Early Maternal-Fetal Interface in Humans, Nature. (2018) 563, no. 7731, 347–353, 10.1038/s41586-018-0698-6, 30429548. PMC7612850 30429548

[4] Zenclussen A. C. and Hämmerling G. J. , Cellular Regulation of the Uterine Microenvironment That Enables Embryo Implantation, Frontiers in Immunology. (2015) 6, 10.3389/fimmu.2015.00321, 26136750. PMC4470084 26136750

[5] Saito S. , Nakashima A. , Shima T. , and Ito M. , Th1/Th2/Th17 and Regulatory T-Cell Paradigm in Pregnancy, American Journal of Reproductive Immunology. (2010) 63, no. 6, 601–610, 10.1111/j.1600-0897.2010.00852.x, 20455873. 20455873

[6] Blanco P. , Palucka A. K. , Pascual V. , and Banchereau J. , Dendritic Cells and Cytokines in Human Inflammatory and Autoimmune Diseases, Cytokine & Growth Factor Reviews. (2008) 19, no. 1, 41–52, 10.1016/j.cytogfr.2007.10.004, 18258476. PMC2413068 18258476

[7] Ziegenhain C. , Vieth B. , Parekh S. , Reinius B. , Guillaumet-Adkins A. , Smets M. , Leonhardt H. , Heyn H. , Hellmann I. , and Enard W. , Comparative Analysis of Single-Cell RNA Sequencing Methods, Molecular Cell. (2017) 65, no. 4, 631–643.e4, 10.1016/j.molcel.2017.01.023. 28212749

[8] Garcia-Alonso L. , Handfield L. F. , Roberts K. , Nikolakopoulou K. , Fernando R. C. , Gardner L. , Woodhams B. , Arutyunyan A. , Polanski K. , Hoo R. , Sancho-Serra C. , Li T. , Kwakwa K. , Tuck E. , Lorenzi V. , Massalha H. , Prete M. , Kleshchevnikov V. , Tarkowska A. , Porter T. , Mazzeo C. I. , van Dongen S. , Dabrowska M. , Vaskivskyi V. , Mahbubani K. T. , Park J. E. , Jimenez-Linan M. , Campos L. , Kiselev V. Y. , Lindskog C. , Ayuk P. , Prigmore E. , Stratton M. R. , Saeb-Parsy K. , Moffett A. , Moore L. , Bayraktar O. A. , Teichmann S. A. , Turco M. Y. , and Vento-Tormo R. , Mapping the Temporal and Spatial Dynamics of the Human Endometrium In Vivo and In Vitro, Nature Genetics. (2021) 53, no. 12, 1698–1711, 10.1038/s41588-021-00972-2, 34857954. PMC8648563 34857954

[9] Stewart C. L. , Kaspar P. , Brunet L. J. , Bhatt H. , Gadi I. , Köntgen F. , and Abbondanzo S. J. , Blastocyst Implantation Depends on Maternal Expression of Leukaemia Inhibitory Factor, Nature. (1992) 359, no. 6390, 76–79, 10.1038/359076a0. 1522892

[10] Chang P. V. , Hao L. , Offermanns S. , and Medzhitov R. , The Microbial Metabolite Butyrate Regulates Intestinal Macrophage Function via Histone Deacetylase Inhibition, Proceedings of the National Academy of Sciences of the United States of America. (2014) 111, no. 6, 2247–2252, 10.1073/pnas.1322269111, 24390544. PMC3926023 24390544

[11] Mohammad S. and Thiemermann C. , Role of Metabolic Endotoxemia in Systemic Inflammation and Potential Interventions, Frontiers in Immunology. (2021) 11, 594150, 10.3389/fimmu.2020.594150, 33505393. PMC7829348 33505393

[12] Squair J. W. , Gautier M. , Kathe C. , Anderson M. A. , James N. D. , Hutson T. H. , Hudelle R. , Qaiser T. , Matson K. J. E. , Barraud Q. , Levine A. J. , la Manno G. , Skinnider M. A. , and Courtine G. , Confronting False Discoveries in Single-Cell Differential Expression, Nature Communications. (2021) 12, no. 1, 10.1038/s41467-021-25960-2, 34584091. PMC8479118 34584091

[13] Crowell H. L. , Soneson C. , Germain P. L. , Calini D. , Collin L. , Raposo C. , Malhotra D. , and Robinson M. D. , Muscat Detects Subpopulation-Specific State Transitions From Multi-Sample Multi-Condition Single-Cell Transcriptomics Data, Nature Communications. (2020) 11, no. 1, 10.1038/s41467-020-19894-4, 33257685. PMC7705760 33257685

